# Molecular Docking and In Silico Predictive Analysis of Potential Herb-Drug Interactions Between Momordica charantia and Miglitol

**DOI:** 10.7759/cureus.84852

**Published:** 2025-05-26

**Authors:** Ponnusankar S, Preethi R, Lithish Kumar MK, Vishal Kesav TR, Harshini VS, Rajesh Kumar R, Balasubramaniam V

**Affiliations:** 1 Department of Pharmacy Practice, JSS College of Pharmacy, JSS Academy of Higher Education and Research, Ooty, IND; 2 Department of Pharmaceutical Biotechnology, Centre of Bioinformatics Research and Advanced Studies, JSS College of Pharmacy, JSS Academy of Higher Education and Research, Ooty, IND; 3 Department of Emergency Medicine, Government Medical College and Hospital, The Nilgiris, Ooty, IND

**Keywords:** alpha-glucosidase inhibitors, charantin, diabetes, herb-drug interaction, molecular docking, momordica charantia

## Abstract

Background

Diabetes mellitus, particularly type 2 diabetes mellitus (T2DM), is a chronic metabolic disorder characterized by persistent hyperglycemia. Alpha-glucosidase inhibitors like miglitol delay carbohydrate absorption, thereby reducing postprandial glucose levels. *Momordica charantia* (bitter melon) has demonstrated hypoglycemic effects in various studies, yet its interactions with pharmaceutical antidiabetic agents remain poorly understood. This study investigates the molecular interactions between *M. charantia* phytoconstituents and miglitol’s enzymatic targets using in silico methods.

Methods

An in silico approach was employed to assess potential herb-drug interactions between *M. charantia* and miglitol. Phytochemical screening identified 18 bioactive compounds from *M. charantia* that complied with Lipinski’s Rule of Five, evaluated using SwissADME. Molecular docking was performed using AutoDock Tools (v1.5.7) to examine binding affinities between these phytoconstituents and key carbohydrate-metabolizing enzymes: lysosomal alpha-glucosidase (GAA), neutral alpha-glucosidases AB (GANAB) and C (GANC), maltase-glucoamylase (MGAM), and pancreatic alpha-amylase (AMY2A). The binding interactions were visualized using PyMOL and LigPlot+ to assess molecular stability.

Results

Molecular docking analysis revealed that charantin exhibited the highest binding affinity across all enzymes, particularly with neutral alpha-glucosidase AB (-12.4 kcal/mol) and maltase-glucoamylase (-12.6 kcal/mol), suggesting strong inhibitory potential. Other phytoconstituents, such as quercetin, luteolin, and kaempferol, also displayed moderate to high affinity, indicating possible synergistic effects. In contrast, compounds like cis-sabinol, myrtenol, and beta-sitosterol showed significantly weaker interactions. The binding interaction analysis confirmed stable hydrogen bonding and hydrophobic interactions between charantin and key enzymatic residues, reinforcing its role as a potent inhibitor of carbohydrate metabolism.

Conclusion

The study suggests that *M. charantia* phytoconstituents, particularly charantin, may enhance miglitol’s effects by inhibiting the same carbohydrate-digesting enzymes. This could lead to increased glucose-lowering efficacy but also raises concerns about excessive inhibition, potentially resulting in postprandial hypoglycemia. These findings underscore the need for careful patient monitoring and dosage adjustments when combining *M. charantia* with alpha-glucosidase inhibitors. While molecular docking provides valuable insights, further in vitro and in vivo studies are essential to validate these computational predictions, assess bioavailability, and determine the clinical implications of *M. charantia-*miglitol co-administration.

## Introduction

Diabetes, primarily type 2 diabetes mellitus (T2DM), is a chronic metabolic condition marked by elevated blood glucose levels, which over the past three decades has seen a significant rise in prevalence across countries of all income levels, leading to potential damage to the heart, blood vessels, eyes, kidneys, and nerves [1]. The diabetes epidemic in India has rapidly escalated from 33 million cases in 2000 to 72 million in 2021, projected to reach 125 million by 2045, with a declining mean age of onset due to genetic predisposition (thin-fat phenotype) and adverse environmental factors [2].

Miglitol, chemically (2R,3R,4R,5S)-1-(2-hydroxyethyl)-2-(hydroxymethyl)piperidine-3,4,5-triol, is one of the widely used second-generation semisynthetic α-glucosidase inhibitors, derived from 1-deoxynojirimycin. It is structurally similar to glucose and is effective in the treatment of T2DM [3]. Miglitol, an α-glucosidase inhibitor, exerts its effect through the delayed absorption of complex carbohydrates in the small intestine, resulting in a decrease in postprandial glucose concentrations that are directly correlated with the dietary carbohydrate content [4]. Clinical trials with Miglitol (usually 50 or 100 mg three times daily) in patients with T2DM consistently demonstrated a significant improvement in glycaemic control for periods of 6 to 12 months [5]. Several clinical trials have evaluated the pharmacodynamic (PD) and pharmacokinetic (PK) properties, bioequivalence, and therapeutic efficacy of Miglitol in glycemic control [6-8]. Both short-term and long-term clinical studies have shown that Miglitol lowers postprandial glucose levels and slightly reduces glycosylated hemoglobin by approximately 0.5-1.0% [4]. Recent estimates indicate that a significant proportion of diabetic patients globally, up to 51%, are utilizing complementary and alternative medicine [9].

*Momordica charantia* L. (*M. charantia*), a member of the Cucurbitaceae family, has traditionally been used as herbal medicine and as a vegetable. The hypoglycemic effects of bitter melon were demonstrated in cell culture, animal models, and human studies [10]. The major compounds isolated from bitter melon and identified as hypoglycemic agents include polysaccharides; proteins and peptides such as polypeptide-p and peroxidase; saponins and terpenoids such as charantin; and flavonoids and phenolic compounds such as quercetin, rutin, kaempferol, and isorhamnetin [11]. One of the major compounds isolated as a saponin from bitter melon and identified as a hypoglycemic agent is charantin, a cucurbitane-type triterpenoid [12,13]. In recent research, charantin was identified as a viable option to treat diabetes and showed the potential to replace conventional treatment entirely [14]. Some evidence has shown that the compound has the capability to be more effective than oral hypoglycemic agents [15].

The hypoglycemic effects of *M. charantia* have been known for years [11], amongst the Indigenous populations of Asia, South America, India, and East Africa [16].

The possibilities of drug interactions, direct toxicities, and contamination with active pharmaceutical agents are among the safety concerns surrounding dietary and herbal supplements. Although there is a widespread public perception that herbs and botanical products in dietary supplements are safe, research has demonstrated that these products carry the same dangers as other pharmacologically active compounds. Interactions may occur between prescription drugs, over-the-counter drugs, dietary supplements, and even small molecules in food, making it a daunting challenge to identify all interactions that are of clinical concern [17]. The result may be either enhanced or diminished drug or herb effects, or the appearance of a new effect that is not anticipated from the use of the drug or herb alone. Although there are several proposed ways to categorize herb-drug interactions, the most logical approach is to characterize interactions from either a PK or PD perspective. Possible PK interactions include those that alter the absorption, metabolism, distribution, or elimination of a drug or herbal constituent, resulting in an increase or decrease in the concentration of the active agent at the site of action [13].

This study investigates the potential herb-drug interactions between *M. charantia* and Miglitol using an in-silico molecular docking approach. Specifically, the study focuses on evaluating the binding interactions between selected phytoconstituents of *M. charantia* and Miglitol’s key enzymatic targets involved in carbohydrate metabolism, namely: lysosomal alpha-glucosidase (GAA), neutral alpha-glucosidases AB (GANAB) and C (GANC), maltase-glucoamylase (MGAM), and pancreatic alpha-amylase (AMY2A). The primary objective of this study is to computationally assess whether bioactive compounds from *M. charantia* exhibit significant binding affinity to these enzymes, thereby indicating a potential for PD herb-drug interactions when co-administered with Miglitol. The study does not evaluate PK interactions and is limited to molecular docking-based predictions, which warrant further in vitro and in vivo validation.

## Materials and methods

The study was conducted at JSS College of Pharmacy, Ooty, which provided all necessary facilities and support for the research. It employed an in silico approach to investigate potential herb-drug interactions between *M. charantia* and Miglitol, an alpha-glucosidase inhibitor used in T2DM management. The methodology included phytochemical screening, drug-likeness evaluation, molecular docking, and binding interaction analysis. As an *in silico* study, no human or animal subjects were involved, eliminating the need for ethical approval. The study adhered to best practices in computational pharmacology to ensure reproducibility and reliability of results.

Ligand preparation

The structures of Miglitol and selected phytoconstituents were obtained from the PubChem database [18]. The selected ligands are listed in Table 1. Energy minimization was performed using Open Babel to optimize ligand conformations. The ligands were prepared for docking by assigning appropriate torsional degrees of freedom and generating PDBQT files in AutoDock Tools (v1.5.7).

**Table 1:** Ligand preparation. The structures of Miglitol and selected phytoconstituents were obtained from the PubChem database. Energy minimization was performed using Open Babel to optimize the ligand conformations. The ligands were then prepared for docking by assigning appropriate torsional degrees of freedom and generating PDBQT files using AutoDock Tools (v1.5.7). IUPAC: International Union of Pure and Applied Chemistry.

S. No.	Ligand Name	IUPAC Name	PubChem CID
1	Myrtenol	(6,6-dimethylbicyclo[3.1.1]hept-2-en-2-yl)methanol	10582
2	Momordicine I	(3S,7S,8S,9R,10R,13R,14S,17R)-3,7-dihydroxy-17-[(2R)-4-hydroxy-6-methylhept-5-en-2-yl]-4,4,13,14-tetramethyl-2,3,7,8,10,11,12,15,16,17-decahydro-1H-cyclopenta[a]phenanthrene-9-carbaldehyde	101293615
3	Charantin	2-(diethylamino)ethyl 2-(naphthalen-1-ylmethyl)-3-(oxolan-2-yl)propanoate; oxalic acid	312915
4	Diosgenin	(1S,2S,4S,5'R,6R,7S,8R,9S,12S,13R,16S)-5',7,9,13-tetramethylspiro[5-oxapentacyclo[10.8.0.0²,⁹.0⁴,⁸.0¹³,¹⁸]icos-18-ene-6,2'-oxane]-16-ol	99474
5	Kaempferol	3,5,7-trihydroxy-2-(4-hydroxyphenyl)chromen-4-one	5280863
6	Quercetin	2-(3,4-dihydroxyphenyl)-3,5,7-trihydroxychromen-4-one	5280343
7	Luteolin	2-(3,4-dihydroxyphenyl)-5,7-dihydroxychromen-4-one	5280445
8	Momordicoside-K	(3S,7S,8S,9R,10R,13R,14S,17R)-3-hydroxy-17-[(E,2R)-6-methoxy-6-methylhept-4-en-2-yl]-4,4,13,14-tetramethyl-7-[(2R,3R,4S,5S,6R)-3,4,5-trihydroxy-6-(hydroxymethyl)oxan-2-yl]oxy...	57330180
9	Vicine	2,4-diamino-5-[(2S,3R,4S,5S,6R)-3,4,5-trihydroxy-6-(hydroxymethyl)oxan-2-yl]oxy-1H-pyrimidin-6-one	135413566
10	Simiarenol	(3R,3aR,5aR,5bS,9S,11aS,11bR,13aS,13bR)-3a,5a,8,8,11b,13a-hexamethyl-3-propan-2-yl-1,2,3,4,5,5b,6,9,10,11,11a,12,13,13b-tetradecahydrocyclopenta[a]chrysen-9-ol	12442794
11	cis-3-Hexen-1-ol	(Z)-hex-3-en-1-ol	5281167
12	Momordicoside-G	(2R,3S,4R,5R,6R)-2-(hydroxymethyl)-6-[[(1R,4S,5S,8R,9R,12S,13S,16S)-8-[(E,2R)-6-methoxy-6-methylhept-4-en-2-yl]-5,9,17,17-tetramethyl-18-oxapentacyclo[...]	91895422
13	Momordicoside-F1	(2R,3S,4S,5R,6R)-2-(hydroxymethyl)-6-[[(1R,4S,5S,8R,9R,12S,13S,16S)-8-[(E,2R)-6-methoxy-6-methylhept-4-en-2-yl]-...]	44445566
14	Momordicoside-F2	(2R,3S,4R,5R,6R)-2-(hydroxymethyl)-6-[[(1R,4S,5S,8R,9R,12S,13S,16S)-8-[(E,2R)-6-hydroxy-6-methylhept-4-en-2-yl]-...]	44445567
15	Momordicoside-I	(2R,3S,4S,5R,6R)-2-(hydroxymethyl)-6-[[(1R,4S,5S,8R,9R,12S,13S,16S)-8-[(E,2R)-6-hydroxy-6-methylhept-4-en-2-yl]-...]	71717036
16	Beta-Sitosterol	(3S,8S,9S,10R,13R,14S,17R)-17-[(2R,5R)-5-ethyl-6-methylheptan-2-yl]-10,13-dimethyl-2,3,4,7,8,9,11,12,14,15,16,17-dodecahydro-1H-cyclopenta[a]phenanthren-3-ol	222284
17	Stigmasterol	(3S,8S,9S,10R,13R,14S,17R)-17-[(E,2R,5S)-5-ethyl-6-methylhept-3-en-2-yl]-10,13-dimethyl-2,3,4,7,8,9,11,12,14,15,16,17-dodecahydro-1H-cyclopenta[a]phenanthren-3-ol	5280794
18	(+)-cis-Sabinol	(1S,3R,5S)-4-methylidene-1-propan-2-ylbicyclo[3.1.0]hexan-3-ol	94147

Phytoconstituent screening and target preparation

Bioactive compounds from *M. charantia* were identified using the Indian Medicinal Plants, Phytochemistry, and Therapeutics (IMMPAT) database [19] and supporting literature on antidiabetic phytochemicals. Initially, 35 phytoconstituents were considered, and 18 compounds were shortlisted for docking based on their compliance with Lipinski’s Rule of Five using SwissADME software [20], to ensure oral bioavailability and favorable PK properties. While we recognize that some known bioactive compounds with poor drug-likeness may have been excluded, the selection was deliberately restricted to those with acceptable drug-likeness to enhance clinical relevance and translational potential. Miglitol's molecular targets were retrieved from the DrugBank database. Compounds were selected based on antidiabetic relevance from literature and the IMMPAT database. SwissADME was used to evaluate drug-likeness. Only those that fully complied with Lipinski’s Rule of Five, i.e., molecular weight < 500 Da, logP < 5, ≤ 5 hydrogen bond donors, and ≤ 10 hydrogen bond acceptors, were included. Compounds failing these criteria were excluded. The selected targets, GAA, GANAB, GANC, MGAM, and AMY2A, were chosen based on their involvement in carbohydrate metabolism and known interaction with alpha-glucosidase inhibitors. PK profiling, including key ADME properties such as GI absorption, blood-brain barrier (BBB) permeability, and CYP enzyme inhibition potential, was performed using SwissADME. 

Molecular docking and binding analysis

Molecular docking was performed using AutoDock Tools (v1.5.7) and AutoDock Vina to evaluate binding affinities between Miglitol, the selected *M. charantia* phytoconstituents, and the identified target enzymes. In this study, binding affinities were interpreted based on docking scores (in kcal/mol). Binding affinity ≤ -10.0 kcal/mol was deemed as high affinity, between -7.0 and -9.9 kcal/mol was deemed as moderate affinity, and less than -6.9 kcal/mol was deemed as low or weak affinity. Blind docking was implemented to allow for an unbiased search of potential binding sites. Proteins were prepared using AutoDock Tools by removing water molecules, adding polar hydrogen atoms, assigning Gasteiger charges, and saving the files in PDBQT format. For each enzyme, the grid box was centered around the predicted active site, and parameters were set to 40×40×40 Å, with center coordinates determined from the known binding domains (e.g., GAA: x = 40.2, y = 62.5, z = 70.1). The exhaustiveness was set to 8 for all runs to balance thoroughness and computational efficiency. Docking protocol validation was performed by redocking the native or literature-reported ligands (when available) into their respective protein structures. The accuracy of docking was confirmed by maintaining root mean square deviation (RMSD) values under 2.0 Å between docked and crystallographic poses. Docking interactions were analyzed based on binding energy scores, where lower (more negative) values indicated stronger affinity. Visualization and interpretation of interactions were carried out using PyMOL v2.4 and LigPlot+ to assess hydrogen bonds and hydrophobic interactions, thereby confirming ligand stability within the active site. No energy minimization was applied to the protein models obtained from Swiss-Model. Miglitol and 18 *M. charantia* phytoconstituents were downloaded in SDF format from PubChem, converted to PDB, and energy-minimized using Open Babel prior to docking.

## Results

Screening of phytoconstituents

The phytoconstituents from the fruit were selected, resulting in a list of 35 compounds. These were then subjected to drug-likeness analysis using SwissADME software, and 18 phytoconstituents that complied with Lipinski’s Rule of Five were shortlisted for further study, as shown in Table 2.

**Table 2:** SwissADME evaluation of phytoconstituents complying with Lipinski’s Rule of Five. BBB: Blood-Brain Barrier; CYP1A2: Cytochrome P450 Family 1 Subfamily A Member 2; CYP2C9: Cytochrome P450 Family 2 Subfamily C Member 9; CYP3A4: Cytochrome P450 Family 3 Subfamily A Member 4; CYP2D6: Cytochrome P450 Family 2 Subfamily D Member 6; N/A: Not Applicable.

S. No.	Phytoconstituent	Drug Likeness	Absorption	Distribution	Metabolism	Excretion
1	Myrtenol	Yes	GI absorption: High	BBB permeability: Yes	N/A	N/A
2	Momordicine I	Yes	GI absorption: High	BBB permeability: No	CYP3A4 inhibitor	P-glycoprotein substrate
3	Charantin	Yes	GI absorption: High	BBB permeability: No	CYP2D6 inhibitor, CYP3A4 inhibitor	P-glycoprotein substrate
4	Diosgenin	Yes	GI absorption: High	BBB permeability: Yes	N/A	N/A
5	Kaempferol	Yes	GI absorption: High	BBB permeability: No	CYP1A2 inhibitor, CYP2D6 inhibitor, CYP3A4 inhibitor	N/A
6	Quercetin	Yes	GI absorption: High	BBB permeability: No	CYP1A2 inhibitor, CYP2D6 inhibitor, CYP3A4 inhibitor	N/A
7	Luteolin	Yes	GI absorption: High	BBB permeability: No	CYP1A2 inhibitor, CYP2D6 inhibitor, CYP3A4 inhibitor	N/A
8	Momordicoside-K	Yes	GI absorption: Low	BBB permeability: No	N/A	N/A
9	Vicine	Yes	GI absorption: Low	BBB permeability: No	N/A	N/A
10	Simiarenol	Yes	GI absorption: Low	BBB permeability: No	N/A	N/A
11	cis-3-Hexen-1-ol	Yes	GI absorption: High	BBB permeability: Yes	N/A	N/A
12	Momordicoside-G	Yes	GI absorption: Low	BBB permeability: No	N/A	P-glycoprotein substrate
13	Momordicoside-F1	Yes	GI absorption: Low	BBB permeability: No	N/A	P-glycoprotein substrate
14	Momordicoside-F2	Yes	GI absorption: Low	BBB permeability: No	N/A	P-glycoprotein substrate
15	Momordicoside-I	Yes	GI absorption: Low	BBB permeability: No	N/A	P-glycoprotein substrate
16	Beta-Sitosterol	Yes	GI absorption: Low	BBB permeability: No	N/A	N/A
17	Stigmasterol	Yes	GI absorption: Low	BBB permeability: No	CYP2C9 inhibitor	N/A
18	(+)-cis-Sabinol	Yes	GI absorption: High	BBB permeability: Yes	N/A	N/A

Selection of drug targets

**Figure 1:**
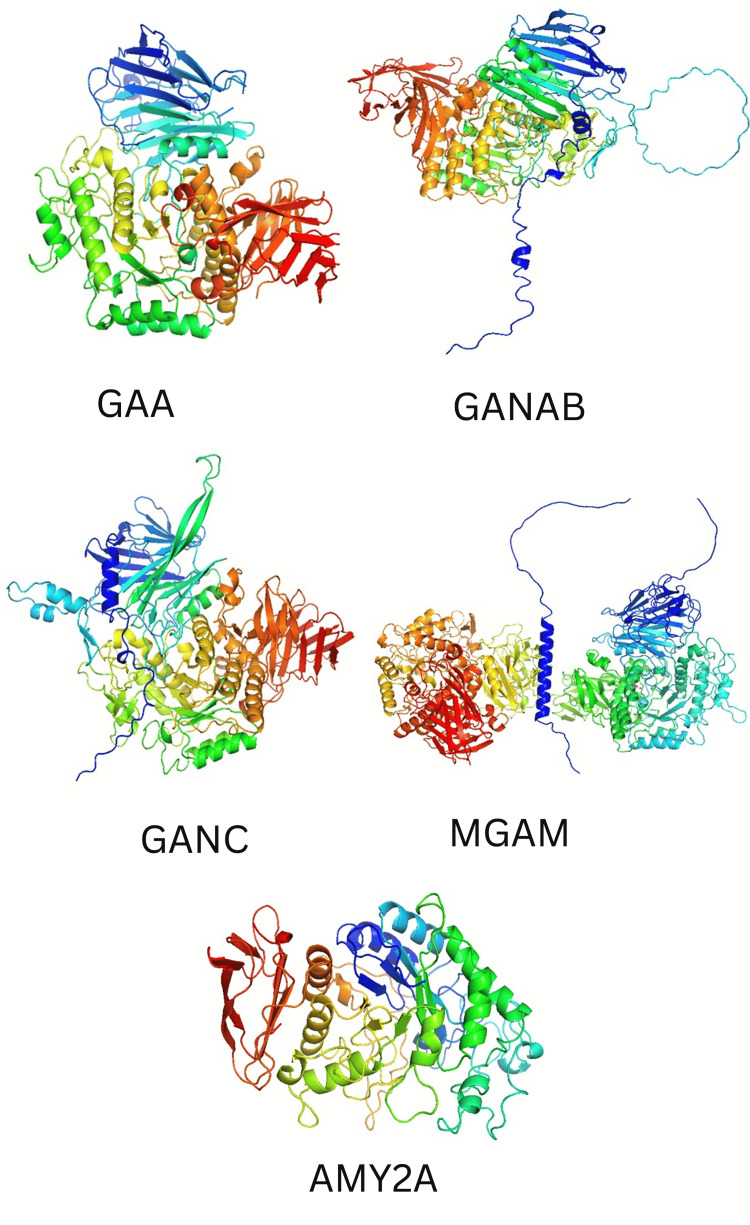
3D structure of proteins predicted using Swiss-Model. These predicted structures were further used for molecular docking, molecular visualization studies, and determination of amino acid binding sites. GAA: Lysosomal alpha-glucosidase; GANAB: Neutral alpha-glucosidase AB; GANC: Neutral alpha-glucosidase C; MGAM: Maltase-glucoamylase; AMY2A: Pancreatic alpha-amylase.

Miglitol exerts its pharmacological activity through GAA, GANAB, GANC, MGAM, and AMY2A, which were selected for molecular docking studies. The 3D structures of GAA, GANAB, GANC, MGAM, and AMY2A were retrieved from the UniProt database, as shown in Figure 1.

Molecular docking and molecular visualization

Most of the phytoconstituents showed high affinity toward the target enzymes. In GAA, 15 phytoconstituents exhibited higher affinity, with Charantin showing the highest binding affinity among them, with a score of -11.9 kcal/mol. Cis-Sabinol produced the least binding affinity, with a score of -5.0 kcal/mol. In GANAB, 17 phytoconstituents showed higher affinity, with Charantin again exhibiting the highest binding affinity at -12.4 kcal/mol, while Myrtenol showed the lowest at -4.6 kcal/mol. In GANC, 15 phytoconstituents demonstrated greater affinity, with Charantin showing the strongest interaction at -12.1 kcal/mol, and Vicine the weakest at -5.6 kcal/mol. In MGAM, 16 phytoconstituents showed higher affinity, with Charantin again showing the strongest interaction at -12.6 kcal/mol, and Myrtenol the weakest at -5.3 kcal/mol. In pancreatic AMY2A, 17 phytoconstituents exhibited greater affinity, with Charantin showing the highest binding score at -12.6 kcal/mol, while Cis-Sabinol and Beta-sitosterol showed the least binding affinity, each with a score of -5.1 kcal/mol. The evaluation of Miglitol-*M. charantia* interactions using AutoDock Tools is depicted in Table 3.

**Table 3:** Evaluation of Miglitol-M. charantia interactions using AutoDock Tools v1.5.7.

Phytoconstituent	Docking Score				
	Lysosomal α-glucosidase (GAA)	Neutral α-glucosidase AB (GANAB)	Neutral α-glucosidase C (GANC)	Maltase-glucoamylase (MGAM)	Pancreatic α-amylase (AMY2A)
Myrtenol	-5.1	-4.6	-7.7	-5.3	-5.3
Momordicine I	-6.8	-6.4	-12.1	-7.3	-7.3
Charantin	-11.9	-12.4	-7.5	-12.6	-12.6
Diosgenin	-7.2	-7.7	-7.2	-7.9	-7.9
Kaempferol	-6.9	-6.6	-7.1	-7.4	-7.4
Quercetin	-6.5	-6.2	-7.1	-7.5	-7.5
Luteolin	-6.8	-7	-7.7	-8.2	-8.2
Momordicoside K	-7.7	-7.1	-5.6	-7.6	-7.6
Vicine	-5.7	-5.5	-7.3	-6.4	-6.4
Simiarenol	-8	-6.9	-6.6	-7.6	-7.6
Momordicoside G	-6.8	-6.7	-8	-7.6	-7
Momordicoside F1	-6.8	-7.2	-7.1	-8.1	-7.3
Momordicoside F2	-8.3	-7	-7.5	-8.3	-8
Momordicoside I	-8	-7.4	-6	-8.9	-7.6
beta-Sitosterol	-6.6	-5.8	-6.4	-6.5	-5.1
Stigmasterol	-6.6	-6		-6.5	-7.7
(+)-cis-Sabinol	-5	-5			-5.1

**Figure 2:**
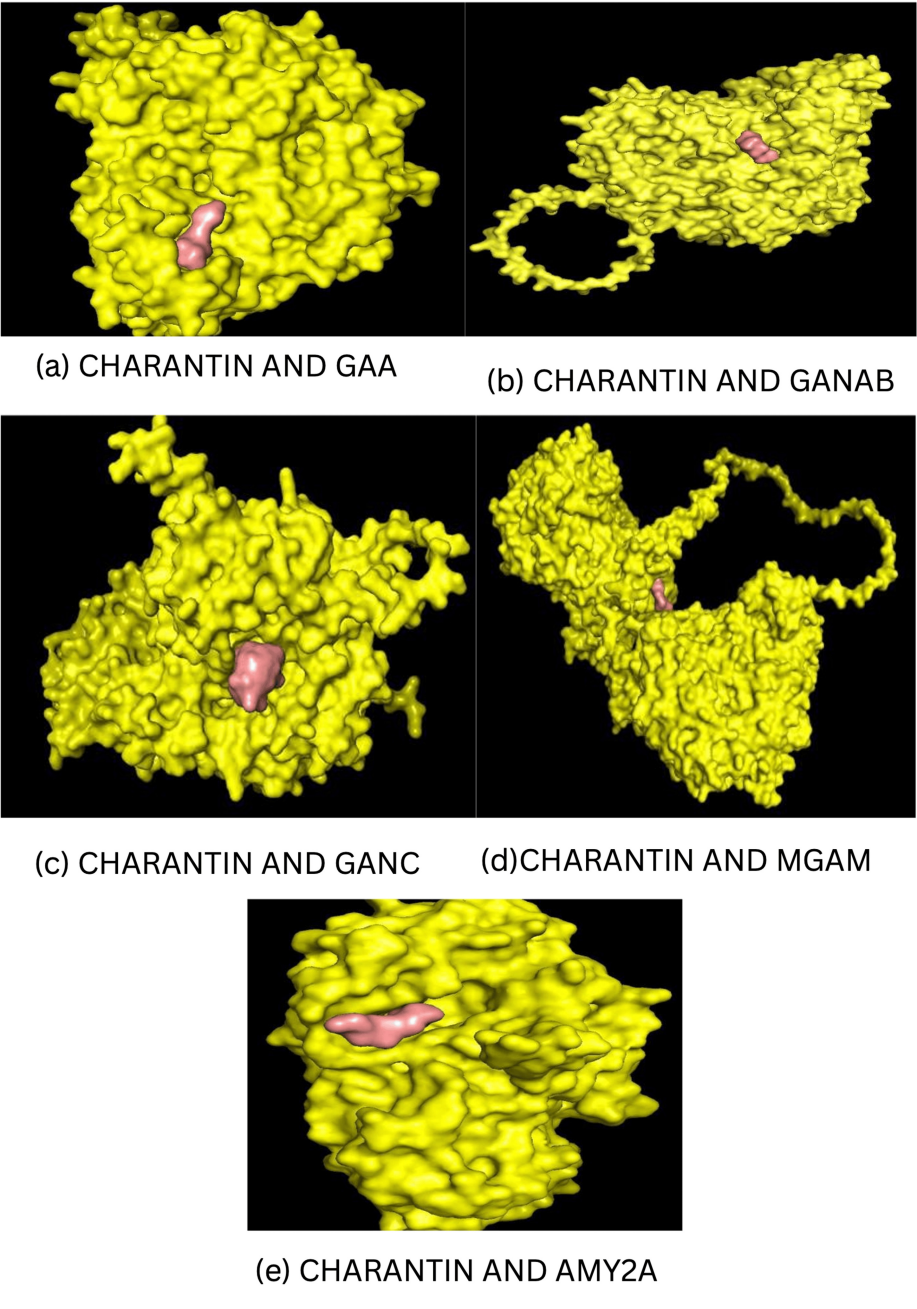
Structural visualization of key binding sites. (a)-(e) confirm the stable binding of phytoconstituents that exhibited maximum affinity in molecular docking studies. GAA: Lysosomal alpha-glucosidase; GANAB: Neutral alpha-glucosidase AB; GANC: Neutral alpha-glucosidase C; MGAM: Maltase-glucoamylase; AMY2A: Pancreatic alpha-amylase.

The evaluation of Miglitol-*M. charantia* interactions was performed using AutoDock Tools and analyzed for molecular docking interactions. The structural visualization of these interactions, highlighting the key binding sites, is illustrated in Figure 2 using PyMOL.

The results suggest that many of these phytoconstituents have the potential to inhibit enzymes critical to glucose metabolism and diabetes management. Among the studied compounds, Charantin consistently exhibited the highest binding affinity across all enzymes. This compound demonstrated remarkable binding scores, particularly with neutral alpha-glucosidase AB (-12.4 kcal/mol) and maltase-glucoamylase (-12.6 kcal/mol), indicating a strong and potentially effective inhibitory action. Charantin's ability to show strong binding across different enzymes highlights its potential as a lead compound for the development of enzyme inhibitors that may be useful in managing hyperglycemia.

In contrast, compounds such as cis-sabinol, Myrtenol, beta-sitosterol, and Vicine showed relatively weaker binding affinities across the various enzymes studied. For instance, cis-sabinol exhibited the lowest affinity toward lysosomal alpha-glucosidase (-5.0 kcal/mol) and pancreatic alpha-amylase (-5.1 kcal/mol). This indicates that while these compounds may possess some inhibitory potential, they are less likely to produce significant enzyme inhibition compared to stronger binders like Charantin.

Overall, the docking results highlight Charantin as a promising candidate for further in vitro and in vivo studies, given its consistent and strong binding affinity across multiple target enzymes. Further studies are necessary to evaluate the PK properties, bioavailability, and therapeutic potential of these compounds in biological systems.

Analysis of binding interaction

**Figure 3:**
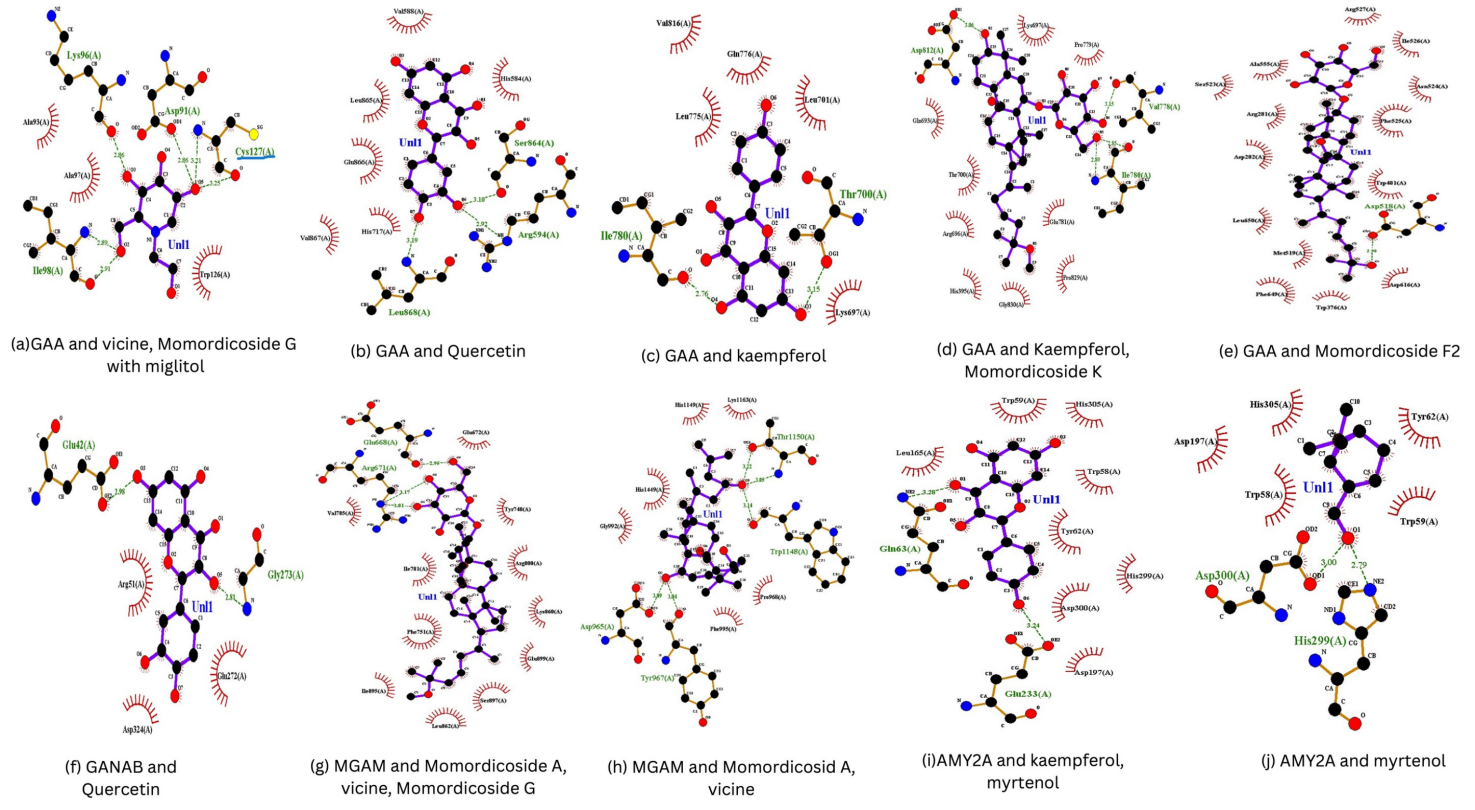
Identified binding sites. The binding sites identified for each ligand–protein interaction are illustrated as follows: (a) Binding interactions of GAA with Vicine, Momordicoside G, and Miglitol. The ligands share similar non-interacting amino acid residues and competitively bind to Cys127(A). (b) Binding interactions of GAA with Quercetin. (c) Binding interactions of GAA with Kaempferol. The ligand competes for Ile780(A) and shares nearly identical non-interacting amino acid residues with Momordicoside K. (d) Binding interactions of GAA with Kaempferol and Momordicoside K. (e) Binding interactions of GAA with Momordicoside F2. (f) Binding interactions of GANAB with Quercetin. (g) Binding interactions of MGAM with Momordicoside A, Vicine, and Momordicoside G. The ligands share similar non-interacting amino acid residues and compete for Glu668(A). (h) Binding interactions of MGAM with Momordicoside A and Vicine. (i) Binding interactions of AMY2A with Kaempferol and Myrtenol. (j) Binding interactions of AMY2A with Myrtenol. GAA: Lysosomal alpha-glucosidase; GANAB: Neutral alpha-glucosidase AB; GANC: Neutral alpha-glucosidase C; MGAM: Maltase-glucoamylase; AMY2A: Pancreatic alpha-amylase; ADME: Absorption, Distribution, Metabolism, and Excretion; IUPAC: International Union of Pure and Applied Chemistry; RMSD: Root Mean Square Deviation; PDB: Protein Data Bank; PDBQT: Protein Data Bank, Partial Charges and Torsions; SDF: Structure Data File.

After docking the ligands to the protein, the results revealed that both the phytoconstituent-enzyme complexes and the Miglitol-enzyme complexes consistently exhibited hydrogen bonding. Figure 3 illustrates the identified binding sites. For lysosomal alpha-glucosidase, the phytoconstituents Vicine, Momordicoside G, and Miglitol share similar non-interacting amino acid residues, as shown in Figure 3a. These ligands exhibit competitive binding for Cys127(A). Similarly, Kaempferol and Momordicoside K share nearly identical non-interacting amino acid residues, as depicted in Figure 3c, competing for Ile780(A). In maltase-glucoamylase, the phytoconstituents Momordicoside A, Vicine, and Momordicoside G also share similar non-interacting amino acid residues, competing for Glu668(A), as illustrated in Figure 3g.

## Discussion

The study highlights the strong inhibitory potential of certain phytoconstituents from *M. charantia* against key enzymes involved in glucose metabolism, emphasizing their potential role in diabetes management. Among the 18 phytoconstituents that complied with Lipinski’s Rule of Five, Charantin consistently exhibited the highest binding affinity across all target enzymes, particularly neutral alpha-glucosidase AB (-12.4 kcal/mol) and maltase-glucoamylase (-12.6 kcal/mol). These findings suggest Charantin as a promising candidate for enzyme inhibition, which could aid in reducing postprandial glucose levels, similar to existing alpha-glucosidase inhibitors like miglitol. Conversely, compounds like cis-sabinol, Myrtenol, and beta-sitosterol showed weaker binding affinities, indicating comparatively lower inhibitory potential. The molecular docking studies also revealed stable hydrogen bonding interactions between phytoconstituents and enzyme active sites, reinforcing their potential efficacy. Clinically, these findings support the exploration of *M. charantia* phytoconstituents as natural alternatives or complementary agents to conventional antidiabetic drugs. The ability of Charantin and other bioactive compounds to modulate key carbohydrate-digesting enzymes suggests their potential use in developing novel therapeutics for glycemic control in diabetic patients.

The findings of our study align with Hussain F et al. [21], demonstrating Charantin’s hypoglycemic effects but providing novel insights into its mechanistic interaction at the molecular level. FTIR analysis revealed distinct functional groups, including C-O, C=C, and C-H stretching, consistent with earlier reports [21]. Peaks were identified by Hussain F et al. [21], detecting alkanes at 2849.5 cm⁻¹ and 1395.9 cm⁻¹, supporting our findings. Furthermore, the presence of carbonyls, as indicated in the 1720-1820 cm⁻¹ range, was also documented, confirming the existence of aldehydes, esters, and ketones in *M. charantia*. Our study further differentiates the binding affinities of other phytoconstituents, highlighting weaker inhibitors such as cis-sabinol, Myrtenol, and beta-sitosterol, which exhibited lower docking scores. Additionally, the identification of key hydrogen bonding interactions supports Charantin’s strong inhibitory potential, reinforcing its role as a promising lead compound for enzyme-targeted diabetes management. These findings underscore the therapeutic relevance of *M. charantia* phytoconstituents and build upon prior research by offering a structure-based perspective on their efficacy.

The study by Gao Y et al. [22] focused on the isolation and characterization of cucurbitane-type triterpene glycosides from *M. charantia* and evaluated their in vitro α-glucosidase inhibitory activity. Their findings showed that certain isolated compounds (2, 5, 7, 8, and 9) exhibited moderate inhibitory activity against α-glucosidase, with IC₅₀ values ranging from 28.40 to 63.26 μM, while acarbose, a known α-glucosidase inhibitor, had an IC₅₀ of 87.65 ± 6.51 μM. In contrast, our study investigated the whole extract of *M. charantia* and its interaction with miglitol, a clinically used α-glucosidase inhibitor. Our results demonstrated a significantly higher inhibitory potency of *M. charantia* extract against α-glucosidase compared to individual triterpene glycosides reported by Gao Y et al. This suggests a possible synergistic effect of multiple bioactive compounds within the extract. Furthermore, our study highlighted the risk of PK interactions when *M. charantia* is co-administered with miglitol, as its strong α-glucosidase inhibition may enhance the glucose-lowering effects, potentially leading to excessive postprandial hypoglycemia.

While our study focused on the interaction between *M. charantia* and miglitol, demonstrating its potent alpha-glucosidase inhibitory activity and potential for drug interactions, Abdollahi M et al. [23] investigated the effects of *M. charantia* aqueous extract on pancreatic histopathology in neonatal Streptozotocin (STZ)-induced diabetic rats. Their findings suggest that *M. charantia* has a regenerative effect on pancreatic β-cells, comparable to glibenclamide, with significant improvements in insulin levels and blood glucose reduction. Clinically, these contrasting mechanisms highlight the multifaceted antidiabetic potential of *M. charantia*, while our study raises concerns about its potential to amplify the effects of miglitol, leading to possible hypoglycemic events, Abdollahi M et al.’s [23] findings suggest its utility in β-cell regeneration, which could be beneficial in progressive diabetes. This underscores the need for careful patient selection when considering *M. charantia* as an adjunct therapy, particularly in those receiving alpha-glucosidase inhibitors, to avoid excessive glucose-lowering effects.

Chokki M et al. [24] demonstrated the strong α-amylase and β-glucosidase inhibitory activities of *M. charantia* and Morinda lucida leaf extracts, attributing these effects to their rich phenolic and flavonoid content. Their findings support the traditional use of these plants in managing postprandial hyperglycemia, suggesting their potential as functional food ingredients or therapeutic agents. Notably, their study emphasized β-glucosidase inhibition as a key mechanism for glucose regulation. In contrast, our study specifically investigated *M. charantia* extract’s potent α-glucosidase inhibition and its interaction with miglitol, a clinically used α-glucosidase inhibitor. Our results highlight a significantly higher inhibitory potency, raising concerns about potential drug interactions and excessive glucose-lowering effects. Unlike Chokki M et al., who focused on general enzyme inhibition and antioxidant properties, our study provided clinically relevant insights into *M. charantia*'s role in diabetes treatment, emphasizing the need for careful use alongside existing antidiabetic medications to avoid hypoglycemia risks.

Our study highlights *M. charantia*’s strong α-glucosidase inhibitory potential, particularly due to Charantin, which exhibited the highest binding affinity among tested phytoconstituents. Compared to previous studies, our findings demonstrate a significantly higher inhibitory potency, suggesting a synergistic effect of multiple bioactive compounds within the extract. However, this raises PK concerns when co-administered with miglitol, a clinically used α-glucosidase inhibitor.

Pharmacologically, *M. charantia* may enhance miglitol’s effects by further inhibiting α-glucosidase, leading to excessive suppression of carbohydrate digestion and absorption. This could result in an increased risk of postprandial hypoglycemia, particularly in patients on combination therapy. Clinically, this interaction necessitates careful patient monitoring, dose adjustments, or potential avoidance in high-risk individuals. While *M. charantia* also exhibits β-cell regenerative properties, its dual role in glucose metabolism suggests both therapeutic benefits and risks. Thus, its integration into diabetes management must be approached cautiously, especially in patients using pharmacological α-glucosidase inhibitors.

While our study included in silico ADME profiling to evaluate PK suitability, it did not incorporate toxicity prediction tools or off-target interaction screening, which are critical components of full ADMET profiling. Furthermore, molecular docking provides only a static view of ligand-protein interactions and does not reflect dynamic biological conditions, such as enzyme flexibility, in vivo bioavailability, or potential adverse effects. As such, future studies should incorporate computational toxicity models and broad-spectrum target prediction to assess safety and off-target risks. Additionally, in vitro and in vivo validation of the predicted binding affinities and pharmacological effects is essential to confirm the therapeutic relevance of these phytoconstituents.

While the molecular docking results indicate that Charantin exhibits strong binding affinity across multiple target enzymes, there are several limitations to consider. First, molecular docking studies only provide theoretical predictions of binding interactions and do not account for dynamic biological factors such as enzyme flexibility, solvent effects, and allosteric modulation. Additionally, factors such as bioavailability, metabolic stability, and toxicity were not assessed in this study. Even if Charantin exhibits strong in silico binding, it may face challenges related to poor absorption, rapid metabolism, or potential adverse effects when administered in a biological system. Future PK and PD studies are essential to determine whether Charantin can be developed into a viable therapeutic agent.

Lastly, this study primarily focused on enzyme inhibition through molecular docking, without exploring potential synergistic or antagonistic interactions with other bioactive compounds present in plant extracts. The complex nature of plant-based treatments means that additional investigations, including combination studies and mechanistic evaluations, are necessary to fully understand their therapeutic potential in diabetes management.

Future research should focus on validating the molecular docking results through in vitro enzyme inhibition assays and in vivo studies to assess Charantin's efficacy in regulating blood glucose levels. Additionally, PK and bioavailability studies are necessary to determine its absorption, distribution, metabolism, and excretion in biological systems. Structure-activity relationship (SAR) studies could further optimize Charantin's potency and specificity, while toxicity and safety evaluations will be crucial to ensure its therapeutic viability. Investigating potential synergistic effects with other compounds and conducting clinical trials in diabetic patients will also be essential steps in determining Charantin’s effectiveness as a natural antidiabetic agent.

## Conclusions

This study provides valuable insights into the strong α-glucosidase inhibitory potential of *M. charantia*, particularly highlighting Charantin as a key bioactive compound with significant enzyme-binding affinity. The molecular docking results indicate that *M. charantia* could serve as a promising adjunct in diabetes management, offering natural inhibition of carbohydrate-digesting enzymes. However, the findings also raise important pharmacological concerns regarding its interaction with miglitol, an established α-glucosidase inhibitor. The strong inhibitory action of *M. charantia* could potentiate the glucose-lowering effects of miglitol, increasing the risk of excessive postprandial hypoglycemia.

This clinically necessitates careful consideration in patients who are already on pharmacological glucose-lowering therapies. The potential herb-drug interaction underscores the need for monitoring blood glucose levels and adjusting dosages accordingly. Further in vitro and in vivo studies are essential to validate these computational findings, assess bioavailability, and evaluate long-term safety. Future research should also explore synergistic effects with other antidiabetic agents and conduct clinical trials to determine the therapeutic viability of *M. charantia*-based interventions.
